# An optimal load indirect matching method without parameter identification and system efficiency optimization

**DOI:** 10.1038/s41598-023-27505-7

**Published:** 2023-01-10

**Authors:** Xueying Qiu, Pan Sun, Enguo Rong, Kangheng Qiao, Jun Sun, Xusheng Wu

**Affiliations:** 1 College of Electrical Engineering, Naval University of Engineering, Wuhan, 430033 China; 2 Department of Mechanical Engineering, Army Artillery and Air Defense Forces Academy, Hefei, 230031 Anhui China

**Keywords:** Electrical and electronic engineering, Energy infrastructure

## Abstract

In some wireless charging applications where the coil spacing varies in real time, such as UAV, electric boat and tram, etc., the traditional direct impedance matching method is difficult to identify the mutual inductance timely and accurately, thus affecting the efficiency optimization effect of the system. In this paper, an indirect impedance matching method without parameter identification is proposed, this method is based on the characteristic that the optimal voltage gain of the resonator is only related to its inherent parameters, and impedance matching can be achieved by controlling the voltage gain in real time. To further improve the efficiency of the system, a single-sided detuning design method is used to achieve soft switching of the inverter. Based on the optimal voltage gain expression derived by using both the indirect impedance matching method and the single-sided detuning design method, a compound control strategy for a series-series-compensated topology with dual-side power control is proposed to improve efficiency and stabilize the output voltage. A hardware prototype is built and a peak DC-to-DC efficiency with the optimal output resistance *R*_L_ at about 28.9 Ω is 91.58%. When the output resistance *R*_L_ is 100 Ω, the efficiency improved by 7% after using the proposed strategy.

## Introduction

Over the past two decades, many studies have described the need to use new energy technologies due to the increase in greenhouse gas emissions and the use of electricity in non-renewable fuels^1,2^. New energy electric vehicles came into being^3,4^, but the charging technology of new energy electric vehicles is a difficult problem. Compared with the traditional conductive charger, the MCR-WPT system has the advantages of flexibility, convenience, safety and reliability^5–8^, because it eliminates the need for charging cables. In addition, wireless power transmission technology is also widely used in underwater equipments, implantable medical equipments, drones and other fields^9–13^.

MCR-WPT system transfers power between the primary coil and the secondary coil, which are magnetically coupled and require the employment of compensation capacitors to resonate with them. The most established topology for MCR-WPT is the series-compensated primary and secondary coil, with the advantages of high resonant frequency stability, low system cost and low control complexity, etc. In literature, the researches on MCR-WPT system mostly focus on analyzing the characterization of this system, especially the efficiency characterization^14–16^.

The overall efficiency of an MCR-WPT system largely depends on the losses that incur in converters and coupling coils. In case of the former, many studies have been reported in relation to the development of converter topologies^17,18^, soft-switching technology^19–21^ and synchronous rectification technology^22^. In case of the latter, studies focus on the optimization of the coil structure^23,24^ and the impedance matching method to maintain efficient operation of the resonator in response to dynamic changes in load and mutual inductance. The conventional direct impedance matching method requires mutual inductance and load identification, which makes the system complicated. The AC equivalent resistance of the rectifier is modulated to an optimal value by using an active rectifier^25^ or a secondary-side cascaded DC–DC converter^26,27^. In addition, a communication link is needed to obtain the information of the secondary side output voltage, so that the inverter or the primary side DC–DC converter can adjust the output voltage. An indirect impedance matching strategy is implemented in^28^ which uses the secondary side cascaded DC–DC converter to control the output voltage and the primary side cascaded DC–DC converter to track the minimum DC input current instead of the maximum efficiency, which indirectly achieves impedance matching. However, this method is a global search method, and the search process is usually slow.

This paper proposes an optimal voltage gain modulation method to minimize the coil losses by controlling the voltage gain in real time. The advantage of this modulation method is that no parameter identification is required, as the optimal voltage gain of the resonator is only related to its inherent parameters. In addition, the inverter power losses are also improved by using single-sided detuning design method. From these analyses, a compound control strategy for a series-series-compensated topology with dual-side power control is proposed to improve efficiency and stabilize the output voltage. Compared with the conventional direct impedance matching strategy, the proposed strategy can achieve impedance matching without parameter identification (*M*, *R*_L_), which simplifies the hardware design and control difficulty of the system. In addition, the system has high operation reliability and good output dynamic characteristics. The analysis is based on a secondary-side cascaded Boost converter and an input DC voltage power control method, which has a wider power control range than the phase-shifted full-bridge method^29^. A hardware laboratory prototype of 100 V output voltage MCR-WPT system is implemented to investigate the behavior of the proposed strategy. The experimental results show the efficacy of the proposed strategy.

## Result

### Prposed topology and single-sided detuning design analysis

#### Proposed topology and working principle

**Figure 1:**
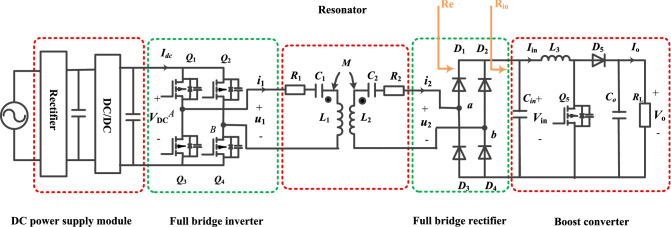
SS-type MCR-WPT system structure with cascaded Boost converter.

**Figure 2:**
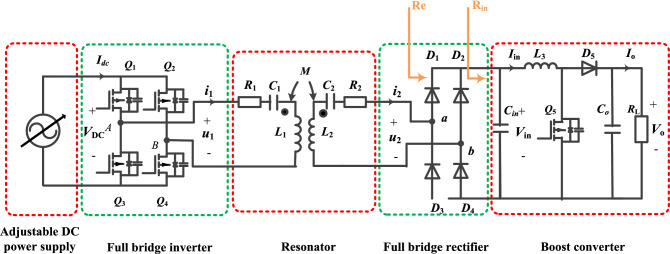
Simplified diagram of system structure.

In Fig. 1,the proposed series-series-compensated MCR-WPT system structure with a secondary-side cascaded Boost converter is depicted, involving DC power supply module, inverter, resonator, rectifier and Boost converter. The DC power supply module rectifies the AC grid voltage into DC, and then outputs the corresponding *V*_DC_ after DC/DC converter as the input voltage of the WPT system. The DC power supply module is not investigated in detail in this paper because it is a standard component and can be designed mostly independently from the other components of the WPT system. The nominal efficiency of the DC power supply module can be assumed to be in the range of 97% to 98%, or even higher for more complex topologies^30,31^. For the convenience of analysis, the AC power grid and DC power supply module at the front end of the inverter are simplified to an adjustable DC power supply, as shown in Fig. 2. Boost converters have simple structure, high reliability and high achievable efficiency^32–34^. The losses of the DC power supply module and Boost converter are no longer considered in this paper. It is worth noting that the DC power supply module and secondary side cascaded Boost converter offer the possibility of implementing the dual target control of this paper. *u*_1_(*i*_1_) is the inverter output voltage(current), *u*_2_(*i*_2_) is the rectifier input voltage (current), *V*_in_ (*I*_*i*n_) is the Boost converter input voltage (current), *V*_o_ (*I*_o_) is the output voltage (current). *R*_L_ is the output resistance, *R*_in_ is the Boost converter input equivalent resistance, *R*_e_ is the rectifier input equivalent resistance, *R*_1_ and *R*_2_ are the internal resistance of the primary coil and secondary coil respectively.

**Figure 3:**
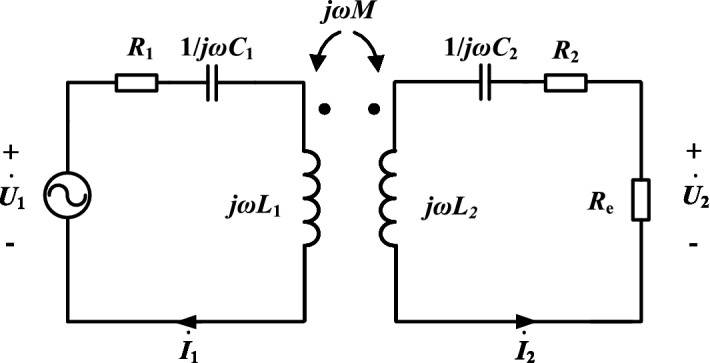
System equivalent circuit phasor model.

To describe the basic behavior of a series-series-compensated WPT system, first-order analysis (FOA) is performed first, and all higher harmonics and losses are neglected^35^. In Fig. 3, *ω* is the operating angular frequency, $\mathop {U_{1} }\limits^{ \bullet }$ is the fundamental phasor of inverter output voltage *u*_1_, $\mathop {U_{2} }\limits^{ \bullet }$ is the fundamental phasor of rectifier input voltage *u*_2_, and $\mathop {I_{1} }\limits^{ \bullet } \left( {\mathop {I_{2} }\limits^{ \bullet } } \right)$ is the fundamental phasor of primary side current (secondary side current).

The system loop voltage equations:

1 $$ \left\{ \begin{aligned} & Z_{11} \mathop {I_{1} }\limits^{ \bullet } + Z_{M} \mathop {I_{2} }\limits^{ \bullet } = \mathop {U_{1} }\limits^{ \bullet } \\ & Z_{M} \mathop {I_{1} }\limits^{ \bullet } + Z_{22} \mathop {I_{2} }\limits^{ \bullet } = \mathop {U_{2} }\limits^{ \bullet } \\ & - R_{e} \mathop {I_{2} }\limits^{ \bullet } = \mathop {U_{2} }\limits^{ \bullet } \\ \end{aligned} \right. $$

where *Z*_11_ and *Z*_22_ are the self-impedances of the primary and secondary sides, *Z*_M_ is the mutual impedance, *Z*_11_ = *R*_1_ + j*ωL*_1_ + 1/j*ωC*_1_, *Z*_22_ = *R*_2_ + j*ωL*_2_ + 1/j*ωC*_2_, *Z*_M_ = j*ωM*.

Solve for the loop currents:

2 $$ \left\{ \begin{gathered} \mathop {I_{1} }\limits^{ \bullet } = - \frac{{\left( {Z_{22} + R_{{\text{e}}} } \right)\mathop {U_{1} }\limits^{ \bullet } }}{{Z_{M}^{2} - Z_{11} \left( {Z_{22} + R_{{\text{e}}} } \right)}} \hfill \\ \mathop {I_{2} }\limits^{ \bullet } = \frac{{Z_{M} \mathop {U_{1} }\limits^{ \bullet } }}{{Z_{M}^{2} - Z_{11} \left( {Z_{22} + R_{{\text{e}}} } \right)}} \hfill \\ \end{gathered} \right. $$

The resonant frequency of the primary side is $1/\sqrt {L_{1} C_{1} }$, and the resonant frequency of the secondary side is $1/\sqrt {L_{2} C_{2} }$. To reduce the reactive power, both the primary and secondary sides are usually in the resonant state, the resonant frequencies of both sides are the same and equal to the working frequency *ω*:

3 $$ \omega =1/\sqrt {L_{1} C_{1} } =1/\sqrt {L_{2} C_{2} } $$

Now, *Z*_11_ = *R*_1_, *Z*_22_ = *R*_2_, *Z*_M_ = j*ωM*, substituting into Eq. (2) to simplify:

4 $$ \left\{ \begin{gathered} \mathop {I_{1} }\limits^{ \bullet } = \frac{{\left( {R_{2} + R_{{\text{e}}} } \right)\mathop {U_{1} }\limits^{ \bullet } }}{{R_{1} \left( {R_{2} + R_{{\text{e}}} } \right){ + }\left( {\omega M} \right)^{2} }} \hfill \\ \mathop {I_{2} }\limits^{ \bullet } = - \frac{{{\text{j}}\omega M\mathop {U_{1} }\limits^{ \bullet } }}{{R_{1} \left( {R_{2} + R_{{\text{e}}} } \right){ + }\left( {\omega M} \right)^{2} }} \hfill \\ \end{gathered} \right. $$

Assuming that $\mathop {U_{1} }\limits^{ \bullet }$ = *U*_1_∠0°,the primary side current $\mathop {I_{1} }\limits^{ \bullet }$ is in phase with the inverter output voltage $\mathop {U_{1} }\limits^{ \bullet }$, the system impedance angle *φ*_1_ is 0°, and the inverter cannot realize soft switching. The coil internal resistance *R*_1_ and *R*_2_ are much smaller than *ωM*, and the RMS of the secondary side current $\mathop {I_{2} }\limits^{ \bullet }$ can be approximated as:

5 $$ I_{2} =\frac{{\omega MU_{1} }}{{R_{1} \left( {R_{2} + R_{{\text{e}}} } \right){ + }\left( {\omega M} \right)^{2} }} \approx \frac{{U_{1} }}{\omega M} $$

When the equivalent resistance *R*_e_ changes, the secondary side current is almost constant. The voltage gain *G*_V_ of the resonator can be approximated as:

6 $$ G_{V} = \left| {\frac{{U_{2} }}{{U_{1} }}} \right| = \left| {\frac{{I_{2} R_{{\text{e}}} }}{{U_{1} }}} \right| \approx \frac{{R_{{\text{e}}} }}{\omega M} $$

The voltage gain *G*_V_ is approximately proportional to the equivalent resistance *R*_e_. The transmission efficiency expression is as follows:

7 $$ \eta = \left| {\frac{{U_{2} I_{2} }}{{U_{1} I_{1} }}} \right| = \frac{{\omega^{2} R_{{\text{e}}} }}{{\left( {R_{{\text{e}}} + R_{2} } \right)\left[ {R_{1} \left( {R_{{\text{e}}} + R_{2} } \right) + \left( {\omega M} \right)^{2} } \right]}} $$

#### Single-sided detuning design

In order to achieve soft switching of the inverter and simplify the analysis, a single-sided detuning design methed is used in this paper. Define the detuning rate *μ*:

8 $$ \mu =1 - \left| {\frac{{Z_{{\text{c}}} }}{{Z_{L} }}} \right|=1 - \frac{1}{{\omega^{2} LC}} $$

where *Z*_C_ and *Z*_L_ are the capacitive reactance and inductive reactance of the detuning side, *Z*_C_ = 1/j*ωC* and *Z*_L_ = j*ωL*. It is necessary to analyze the sensitivity of the system impedance angle to the disturbance of the detuning rate *μ*, as well as the influence when subjected to a wide range of load changes. In addition, the constant current characteristics of the secondary side should also be maintained as much as possible.

When the primary side is resonant and the secondary side is detuned, the inverter operating frequency *ω* is equal to the primary side resonance frequency:

9 $$ \omega =1/\sqrt {L_{1} C_{1} } $$

Now, *Z*_11_ = *R*_1_, *Z*_22_ = *R*_2_ + j*ωL*_2_*μ*, *Z*_M_ = j*ωM*, substituting into Eq. (2) to simplify:

10 $$ \left\{ \begin{gathered} \mathop {I_{1} }\limits^{ \bullet } = \frac{{\left( {R_{2} + {\text{j}}\omega L_{2} \mu + R_{{\text{e}}} } \right)\mathop {U_{1} }\limits^{ \bullet } }}{{R_{1} \left( {R_{2} + R_{{\text{e}}} } \right){ + }\left( {\omega M} \right)^{2} + {\text{j}}\omega L_{2} R_{1} \mu }} \hfill \\ \mathop {I_{2} }\limits^{ \bullet } = - \frac{{{\text{j}}\omega M\mathop {U_{1} }\limits^{ \bullet } }}{{R_{1} \left( {R_{2} + R_{{\text{e}}} } \right){ + }\left( {\omega M} \right)^{2} + {\text{j}}\omega L_{2} R_{1} \mu }} \hfill \\ \end{gathered} \right. $$

The system input impedance angle *φ*_1_ is:

11 $$ \phi_{1} =\angle \left( {\arctan \frac{{\omega L_{2} R_{1} \mu }}{{R_{1} \left( {R_{2} + R_{{\text{e}}} } \right){ + }\left( {\omega M} \right)^{2} }} - \arctan \frac{{\omega L_{2} \mu }}{{R_{2} + R_{{\text{e}}} }}} \right) $$

And the RMS of the secondary current $\mathop {I_{2} }\limits^{ \bullet }$ can be approximated as:

12 $$ I_{2} = \frac{{\omega MU_{1} }}{{\sqrt {\left[ {R_{1} \left( {R_{2} + R_{{\text{e}}} } \right){ + }\left( {\omega M} \right)^{2} } \right]^{2} { + }\left[ {\omega L_{2} R_{1} \mu } \right]^{2} } }} \approx \frac{{U_{1} }}{\omega M} $$

**Figure 4:**
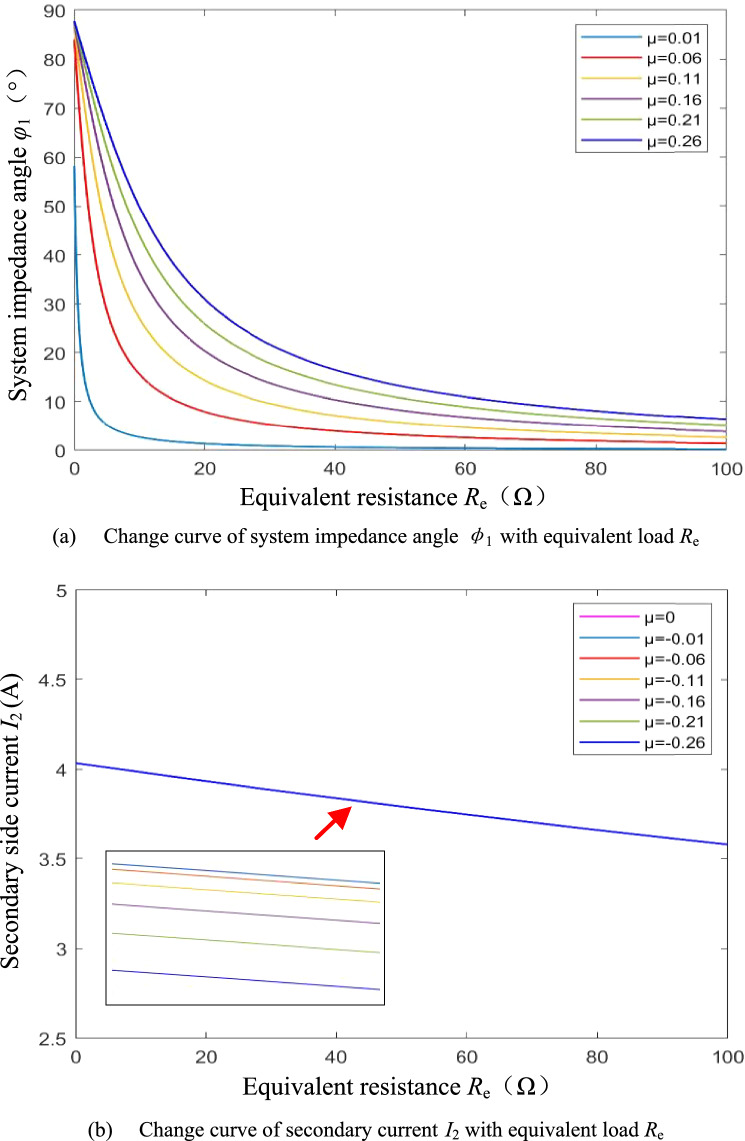
System impedance angle and secondary current when the secondary side is detuned.

From Eq. (11), it can be seen that when detuning rate *μ* < 0, the system impedance angle *φ*_1_ > 0 and the input port of the resonator is inductive. When *R*_e_ is large, since the *ωL*_2_*R*_1_ component is relatively small, the detuning rate *μ* and the equivalent resistance *R*_e_ have non-direct effects on the system impedance angle*φ*_1_, and *φ*_1_ is little sensitive to the detuning rate *μ* perturbation and is little affected by the wide range of variations of the equivalent resistance *R*_e_. Further, since the coil internal resistance *R*_1_, *R*_2_ and detuning rate *μ* are all of order 10^–1^, which is much smaller than *ωM*, according to Eq. (12), the detuning rate *μ* has little effect on the secondary current *I*_2_, and the secondary side is still approximately constant current. Figure 4 shows the change curve of the system impedance angle *φ*_1_ and the secondary side current *I*_2_ with the equivalent resistance *R*_e_ under different detuning ratios *μ* when the secondary side is detuned.

The voltage gain *G*_V_ can be approximated as:

13 $$ G_{V} = \left| {\frac{{U_{2} }}{{U_{1} }}} \right| = \left| {\frac{{I_{2} R_{{\text{e}}} }}{{U_{1} }}} \right| \approx \frac{{R_{{\text{e}}} }}{\omega M} $$

The voltage gain *G*_V_ is still approximately proportional to the equivalent resistance *R*_e_ under the secondary side detuning design. The transmission efficiency expression is as follows:

14 $$ \eta = \frac{{\left( {\omega M} \right)^{2} R_{{\text{e}}} }}{{\sqrt {\left( {R_{{\text{e}}} + R_{2} } \right)^{2} + \left( {\omega L_{2} \mu } \right)^{2} } \sqrt {\left[ {R_{1} \left( {R_{{\text{e}}} + R_{2} } \right) + \left( {\omega M} \right)^{2} } \right]^{2} + \left( {\omega L_{2} R_{1} \mu } \right)^{2} } }} $$

When the primary side is detuned and the secondary side is resonant, the inverter operating frequency *ω* is equal to the secondary resonant frequency:

15 $$ \omega =1/\sqrt {L_{2} C_{2} } $$

Now, *Z*_11_ = *R*_1_ + j*ωL*_1_*μ*, *Z*_22_ = *R*_2_, *Z*_M_ = j*ωM*, substituting into Eq. (2) to simplify:

16 $$ \left\{ \begin{gathered} \mathop {I_{1} }\limits^{ \bullet } = \frac{{\left( {R_{{2}} + R_{{\text{e}}} } \right)\mathop {U_{1} }\limits^{ \bullet } }}{{R_{1} \left( {R_{2} + R_{{\text{e}}} } \right){ + }\left( {\omega M} \right)^{2} + {\text{j}}\omega L_{1} \left( {R_{{2}} + R_{{\text{e}}} } \right)\mu }} \hfill \\ \mathop {I_{2} }\limits^{ \bullet } = - \frac{{{\text{j}}\omega M\mathop {U_{1} }\limits^{ \bullet } }}{{R_{1} \left( {R_{2} + R_{{\text{e}}} } \right){ + }\left( {\omega M} \right)^{2} + {\text{j}}\omega L_{1} \left( {R_{{2}} + R_{{\text{e}}} } \right)\mu }} \hfill \\ \end{gathered} \right. $$

The system input impedance angle *φ*_1_ is:

17 $$ \phi_{1} =\angle \arctan \frac{{\omega L_{1} \left( {R_{2} + R_{{\text{e}}} } \right)\mu }}{{R_{1} \left( {R_{2} + R_{{\text{e}}} } \right){ + }\left( {\omega M} \right)^{2} }} $$

And the RMS of the secondary current $\mathop {I_{2} }\limits^{ \bullet }$ is:

18 $$ I_{2} = \frac{{\omega MU_{1} }}{{\sqrt {\left[ {R_{1} \left( {R_{2} + R_{{\text{e}}} } \right){ + }\left( {\omega M} \right)^{2} } \right]^{2} { + }\left[ {\omega L_{1} \left( {R_{2} + R_{{\text{e}}} } \right)\mu } \right]^{2} } }} $$

**Figure 5:**
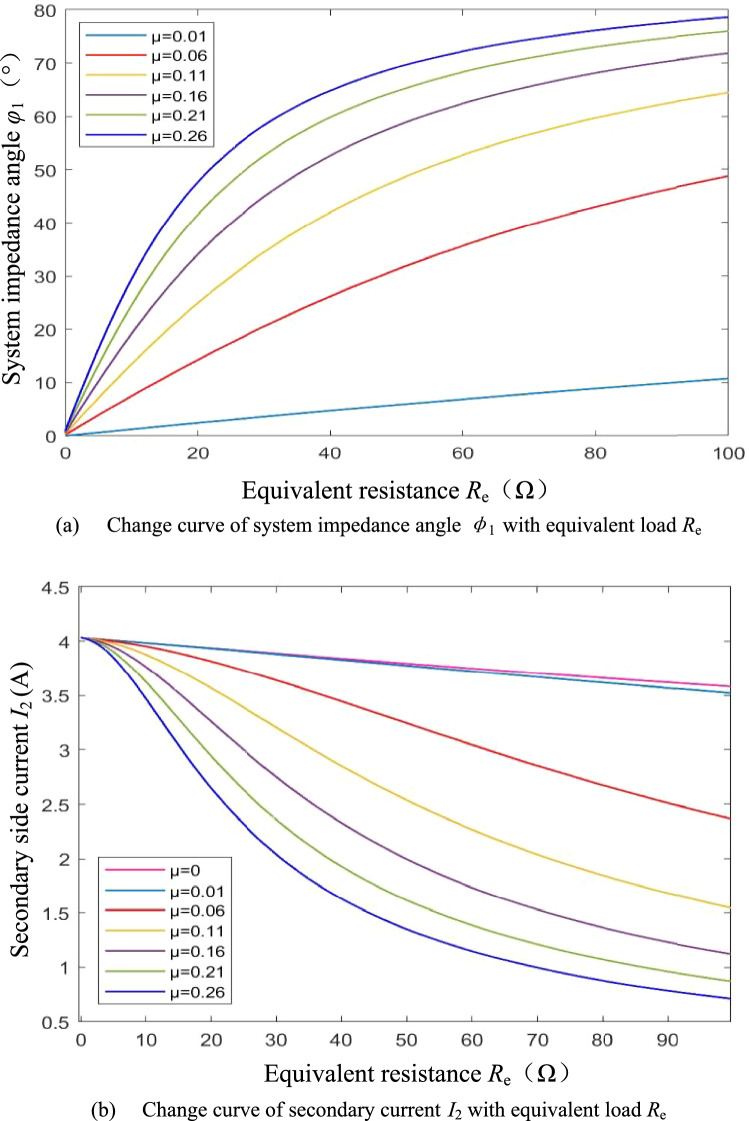
System impedance angle and secondary current when the primary side is detuned.

It can be seen from Eq. (17) that under the primary side detuning design, when the detuning rate *μ* > 0, the system impedance angle *φ*_1_ > 0. Since the *ωL*_1_ (*R*_2_ + *R*_e_) component is large, a slight Δ*μ* can cause a large change in *φ*_1_. The system impedance angle *φ*_1_ is very sensitive to the disturbance of the detuning rate *μ* and is greatly affected by the change of the equivalent load *R*_e_. Analysis of Eq. (18) shows that, again, due to the large component *ωL*_1_ (*R*_e_ + *R*_2_), the sensitivity of the secondary current *I*_2_ to the detuning rate *μ* perturbation is high, and the change of equivalent resistance *R*_e_ also has a large effect on *I*_2_. Figure 5 shows the change curve of the system impedance angle*φ*_1_ and the secondary side current *I*_2_ with the equivalent resistance *R*_e_ under different detuning ratios *μ* when the primary side is detuned.

Comparing Figs. 4 and 5, combined with the above theoretical analysis, there are conclusions: (1) Under the same equivalent resistance *R*_e_, |*μ*| increases, |*φ*_1_| increases, and the system reactive component increases, so |*μ*| should be as small as possible under the premise that soft switching can be achieved within the range of load variation. (2) Under the secondary side detuning design, the system impedance angle *φ*_1_ can be weakly inductive over a larger range of equivalent resistance variations, and is less affected by equivalent resistance variations and detuning rate disturbances, making the soft switching characteristics more stable. (3) Under the secondary side detuning design, the secondary current *I*_2_ is less affected by the detuning rate *μ*. When the internal resistance *R*_1_ of the primary coil is very small, the secondary current is approximately constant. In summary, this paper adopts a secondary side detuning design with better soft switching characteristics and secondary current characteristics. In addition, when the parameters of the primary side cannot be accurately configured, the primary side can be resonated by fine-tuning the operating frequency of the system.

### Maximum efficiency tracking principle and compound control strategy

#### Maximum efficiency tracking principle

From the expression (7) for the transmission efficiency at full resonance on both sides, as *η* is a function of *R*_e_, it can be maximized by adjusting the equivalent resistance *R*_e_ to the optimal value ${\mathop R\limits^{ \circ }}_{{{\text{e-opt}}}}$^19^:

19 $$ \frac{d\eta }{{dR_{{\text{e}}} }}{ = 0} \Rightarrow {\mathop R\limits^{ \circ }}_{{{\text{e-opt}}}} = \sqrt {R_{2}^{2} + \frac{{R_{2} }}{{R_{1} }}\left( {\omega M} \right)^{2} } \approx \sqrt {\frac{{R_{2} }}{{R_{1} }}} \omega M $$

Substituting ${\mathop R\limits^{ \circ }}_{{{\text{e-opt}}}}$ into the approximate expression (6) of voltage gain, the optimal voltage gain ${\mathop G\limits^{ \circ }}_{{{\text{v-opt}}}}$ at full resonance on both sides is obtained:

20 $$ {\mathop G\limits^{ \circ }}_{{V{\text{-opt}}}} \approx \frac{{{\mathop R\limits^{ \circ }}_{{{\text{e-opt}}}} }}{\omega M} \approx \sqrt {\frac{{R_{2} }}{{R_{1} }}} $$

It can be seen that when both sides are resonant, the transmission efficiency *η* reaches the maximum when the equivalent resistance is the optimal value ${\mathop R\limits^{ \circ }}_{{{\text{e-opt}}}}$. The value of ${\mathop R\limits^{ \circ }}_{{{\text{e-opt}}}}$ is not only related to the fixed parameter of the resonator, but also to the variable parameter of mutual inductance *M*. But the optimal voltage gain ${\mathop G\limits^{ \circ }}_{{{\text{v-opt}}}}$ at maximum transmission efficiency is only related to the fixed parameters (*R*_1,_ *R*_2_) of the resonator.

From the expression (14) for the transmission efficiency *η* under the secondary side detuning design is also a function of *R*_e_, since the expression of *R*_e-opt_ is too complicated, the relationship between the transmission efficiency *η*, the optimal equivalent resistance *R*_e-opt_ and the detuning rate *μ* is analyzed according to the data in Table 1.

**Table 1:** System parameters.

Parameters	Values
Resonant frequency *f* (kHz)	84.4
Primary coil inductance (μH)	247.2
Secondary coil inductance (μH)	91.3
Mutual inductance (μH)	46.72
Coupling coefficient *k*	0.31
Primary coil resistance *R*_1_	0.78
Secondary coil resistance *R*_2_	0.30
Primary compensation capacitor *C*_1_	14.4
Secondary compensation capacitor *C*_2_	–

**Figure 6:**
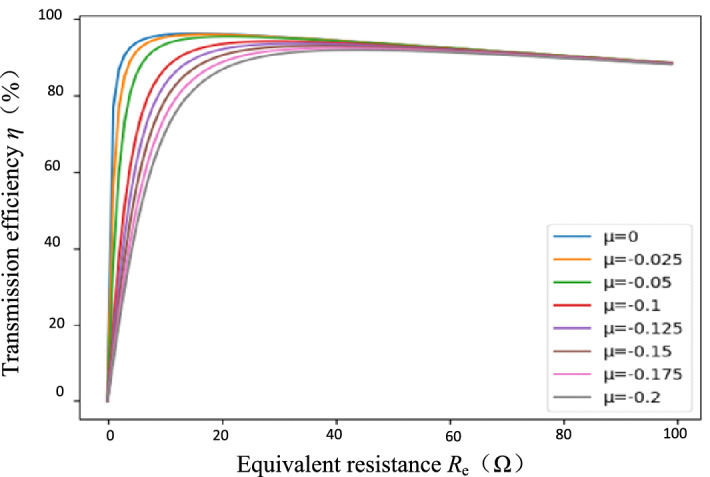
Efficiency–equivalent resistance curve under different detuning ratio *μ.*

As shown in Fig. 6, the *η*–*R*_e_ curves can be visually examined under different detuning rate *μ*(*μ* < 0) when the secondary side is detuned. The larger the |*μ*|, the larger the *R*_e-opt_, and the smaller the corresponding maximum transmission efficiency *η*_max_.

The detuning design will sacrifice resonator efficiency while realizing soft switching. Therefore, under the premise of realizing soft switching within the range of resistance variation, |*μ*| should be as small as possible so that the maximum efficiency *η*_max_ of the resonator can be as large as possible. The secondary side is detuned with *μ* < 0, so *μ* is taken between [− 0.2, 0] at 0.01 intervals. In the following, we will investigate the relationship between the optimal equivalent resistance *R*_e-opt_ and the optimal voltage gain *G*_V-opt_ with respect to the detuning rate* μ* under the secondary side detuning design. Table 2 shows the *R*_e-opt_ values under different *μ.*

**Table 2:** Optimal equivalent resistance *R*_e-opt_ under different detuning rate *μ.*

*μ*	*R*_e-opt_ (Ω)	*μ*	*R*_e-opt_ (Ω)
0	15.36	− 0.11	30.84
− 0.01	15.73	− 0.12	32.37
− 0.02	16.71	− 0.13	33.86
− 0.03	18.06	− 0.14	35.34
− 0.04	19.60	− 0.15	36.79
− 0.05	21.21	− 0.16	38.21
− 0.06	22.85	− 0.17	39.61
− 0.07	24.49	− 0.18	41.00
− 0.08	26.12	− 0.19	42.36
− 0.09	27.72	− 0.2	43.70
− 0.1	29.29		

**Figure 7:**
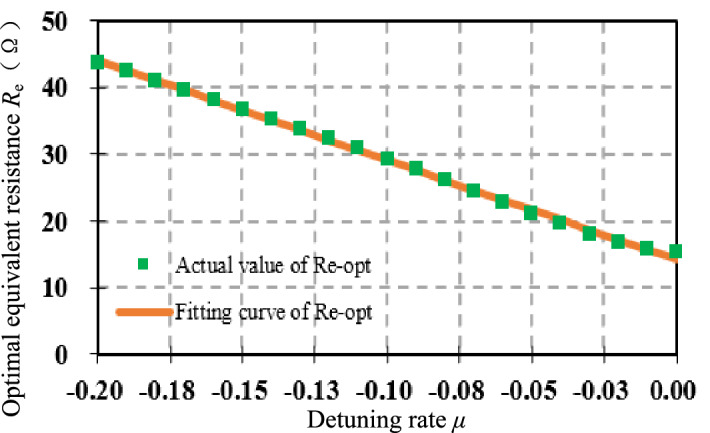
Linear fitting curve of *R*_e-opt_ with respect to *μ.*

Plot the scatter Fig. 7, the relationship between *R*_e-opt_ and *μ* is approximately linear. In fact, it has been verified that after changing the system parameters in Table 1, such as the values of *L*_1_(*L*_2_), *R*_1_(*R*_2_), and *M*, there is still an approximate linear relationship between the optimal equivalent resistance *R*_e-opt_ and the detuning rate *μ*, which will not be repeated here.

The expression fitted using the linear model is given by:

21 $$ {{\mathop R\limits^{ * }}}_{{{\text{e-opt}}}} = - {149}{\text{.56}}\mu { + 14}{\text{.15}}\;(\Omega ) $$

Assuming full resonance on both sides, the actual value ${\mathop R\limits^{ \circ }}_{{{\text{e-opt}}}}$ of the optimal equivalent resistance is equal to the fitted value ${\mathop R\limits^{*}}_{{{\text{e-opt}}}} |_{\mu = 0}$.

22 $$ {\mathop R\limits^{ \circ }}_{{{\text{e-opt}}}} ={\mathop R\limits^{*}}_{{{\text{e-opt}}}} \left| {_{{\mu =0}} } \right.{ = 14}{\text{.15}}\;(\Omega ) $$

Then the fitting value ${\mathop R\limits^{*}}_{{{\text{e-opt}}}}$ of the optimal equivalent resistance under different detuning rate *μ* has the following relationship with the actual value ${\mathop R\limits^{ \circ }}_{{{\text{e-opt}}}}$ at full resonance on both sides:

23 $$ \frac{{{\mathop R\limits^{ * }}_{{{\text{e-opt}}}} }}{{{\mathop R\limits^{ \circ }}_{{{\text{e-opt}}}} }}=\frac{{ - {149}{\text{.56}}\mu { + }14.15}}{14.15} \Rightarrow {{\mathop R\limits^{ * }}}_{{{\text{e-opt}}}} =(1 - 10.5695\mu ) \times {\mathop R\limits^{ \circ }}_{{{\text{e-opt}}}} $$

Combined with the analytical expression (19) of ${\mathop R\limits^{ \circ }}_{{{\text{e-opt}}}}$, the analytical expression of ${\mathop R\limits^{*}}_{{{\text{e-opt}}}}$ under different *μ* is obtained:

24 $$ {{\mathop R\limits^{ * }}}_{{{\text{e-opt}}}} =(1 - 10.5695\mu ) \times \sqrt {\frac{{R_{2} }}{{R_{1} }}} \omega M $$

Substituting expression (24) into the voltage gain approximation expression (13) under the secondary side detuning design, the optimal voltage gain ${\mathop G\limits^{ * }}_{{{\text{v-opt}}}}$ expression under different *μ* is obtained:

25 $$ {\mathop G\limits^{ * }}_{{V{\text{-opt}}}} \approx \frac{{{\mathop R\limits^{ * }}_{{{\text{e-opt}}}} }}{\omega M}=(1 - 10.5695\mu ){*}\sqrt {\frac{{R_{2} }}{{R_{1} }}} $$

It can be seen that the secondary side detuning design has the same law as the full resonance design on both sides when the transmission efficiency is maximum—the corresponding optimal equivalent resistance is not only related to the fixed parameters of the sysytem (*R*_1_, *R*_2_, *μ*, *ω*), but also to the variable parameter of mutual inductance *M*; and the optimal voltage gain is only related to the fixed parameters of the resonator (*R*_1_, *R*_2_, *μ*), thus obtaining the basis for impedance matching.

The traditional direct impedance matching technology uses a secondary side converter or an active rectifier to modulate the equivalent resistance to the optimal value ${\mathop R\limits^{*}}_{{{\text{e-opt}}}}$, which needs to identify the mutual inductance *M* and the load *R*_L_. This paper indirectly realizes impedance matching by controlling the voltage gain to the optimal value ${\mathop G\limits^{ * }}_{{{\text{v-opt}}}}$ in real time, without mutual inductance and load identification.

#### Compound control analysis of voltage regulation and maximum efficiency tracking

In practical applications, in addition to maximum efficiency tracking, the output performance requirements should also be guaranteed. In this paper, the inverter is kept on 180° complementary conduction and two control variables are provided using an adjustable DC power supply and a secondary-side cascaded Boost converter.

In the traditional direct impedance matching strategy, the secondary side Boost converter is used to modulate the equivalent resistance *R*_e_ to the optimal value ${\mathop R\limits^{*}}_{{{\text{e-opt}}}}$, which needs to identify the mutual inductance *M* and the load *R*_L_, the system hardware design and control are complex, and the identification accuracy is difficult to ensure, which further affects the optimization effect of the system efficiency, and the primary side adjustable DC power supply is used to output voltage stabilization, When the communication between the primary side and the secondary side fails, the whole system will no longer work safely. In this paper, the secondary side Boost converter is used to output voltage stabilization, and the the primary side adjustable DC power supply is used for coordinated control to make the voltage gain an optimal value ${\mathop G\limits^{ * }}_{{{\text{v-opt}}}}$ to indirectly realize impedance matching.

The relationship between the RMS value *U*_1_ of the inverter output voltage $\mathop {U_{1} }\limits^{ \bullet }$ and the value *V*_DC_ of the adjustable DC power supply can be expressed as:

26 $$ U_{1} =\frac{2\sqrt 2 }{\pi }V_{DC} $$

Similarly, the relationship between the RMS value *U*_2_ of the rectifier input voltage $\mathop {U_{2} }\limits^{ \bullet }$ and the Boost converter input voltage *V*_in_ can be expressed as:

27 $$ U_{2} =\frac{2\sqrt 2 }{\pi }V_{{{\text{in}}}} $$

Control the *V*_DC_ to make the voltage gain the optimal value ${\mathop G\limits^{ * }}_{{{\text{v-opt}}}}$ [Eq. (25)]:

28 $${\mathop G\limits^{*}}_{{{\text{v-opt}}}} =\frac{{U_{2} }}{{U_{1} }}=\frac{{V_{{{\text{in}}}} }}{{V_{DC} }} \Rightarrow V_{DC} =\frac{{V_{{{\text{in}}}} }}{{\mathop G\limits^{*}_{{{\text{v-opt}}}} }} $$

The change of the adjustable DC source *V*_DC_ will further affect the output voltage *V*_o_. Combining with the voltage transformation relationship of Boost, the relationship between *V*_o_ and *V*_DC_ and the Boost duty cycle *D* is obtained:

29 $$ V_{o} =\frac{1}{1 - D}V_{in} =\frac{1}{1 - D}V_{DC}{\mathop G\limits^{*}}_{{V{\text{-opt}}}} \in \left[ {V_{DC}{\mathop G\limits^{*}}_{{V{\text{-opt}}}} ,\infty } \right) $$

Obviously, the output voltage *V*_o_ is not only affected by the control variable *V*_DC_, but also by the duty cycle *D*.

The duty cycle *D* of the Boost converter can not only transform the voltage, but also transform the impedance. Combining the impedance transformation of the rectifier and Boost converter, the relationship between the rectifier input equivalent resistance *R*_e_ and the output resistance *R*_L_ can be obtained as follows:

30 $$ R_{e} =\frac{8}{{\pi^{2} }}R_{{{\text{in}}}} =\frac{8}{{\pi^{2} }}\left( {1 - D} \right)^{2} R_{L} \in \left( {0,\frac{8}{{\pi^{2} }}R_{L} } \right] $$

Based on the foregoing analysis, the transmission efficiency is only affected by the equivalent resistance *R*_e_ (Namely, the duty cycle *D*), so the voltage regulation control and the maximum efficiency tracking control can operate independently and decoupled from each other.

When the resonator voltage gain is optimal, the transmission efficiency is the maximum, and the equivalent resistance must also be the optimal value ${\mathop R\limits^{*}}_{{{\text{e-opt}}}}$ [Eq. (24)], combined with the Eq. (30), the duty cycle *D*_∞_ of the Boost converter at maximum efficiency is

31 $$ D_{\infty } { = 1} - \sqrt {\frac{{\pi^{2} }}{8}\frac{{{\mathop R\limits^{*}}_{{{\text{e-opt}}}} }}{{R_{L} }}} ,R_{L} \in \left[ {\frac{{\pi^{2} }}{8}{\mathop R\limits^{*}}_{{{\text{e-opt}}}} ,\infty } \right) $$

Substituting *D*_∞_ into Eq. (29), the adjustable DC source *V*_DC∞_ when the output voltage is regulated as *V*_o-ref_ is:

32 $$ V_{DC\infty } =\frac{{V_{{\text{o - ref}}} (1 - D_{\infty } )}}{{{\mathop G\limits^{*}}_{{V{\text{-opt}}}} }} $$

**Figure 8:**
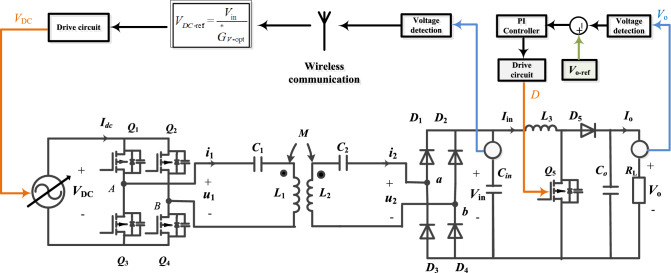
System control loop diagram.

Figure 8 is a compound control loop diagram of voltage regulation and maximum efficiency tracking. When the output resistance *R*_L_ changes, the collected output voltage *V*_o_ information is sent to the secondary controller to regulate the duty cycle *D* of Boost to make the output voltage stabilized as the reference value *V*_o-ref_. The equivalent resistance *R*_e_ varies with *D*. Since the SS-type resonator is a constant-current topology on the secondary side, the voltage gain must deviate from the optimal value ${\mathop G\limits^{ * }}_{{{\text{v-opt}}}}$. Using wireless communication devices, the collected Boost input voltage information *V*_in_ is sent to the primary controller to regulate the adjustable DC power supply *V*_DC_ so that the voltage gain is the optimal value ${\mathop G\limits^{ * }}_{{{\text{v-opt}}}}$. And the change of *V*_DC_ will affect *V*_o_, so continue to regulate the duty cycle *D* to achieve voltage stabilization and regulate the adjustable DC power supply *V*_DC_ to achieve optimal voltage gain ${\mathop G\limits^{ * }}_{{{\text{v-opt}}}}$, and repeat the cycle continuously. When the output resistance *R*_L_ satisfies the boundary conditions of the system control:

33 $$ R_{L} \ge \frac{{\pi^{2} }}{8}{\mathop R\limits^{*}}_{{{\text{e-opt}}}} $$

This reciprocal dynamic coordinated control process eventually converges to a steady state, with *D* and *V*_DC_ converging to the steady state values *D*_∞_ and *V*_DC∞_, respectively [see Eqs. (31) and (32)].

Obviously, compared with the traditional direct impedance matching strategy, the process of using the secondary-side cascaded Boost converter to stablilize the output voltage does not depend on communication equipments, and the system has high operating reliability and good output dynamic characteristics. The process of regulating the adjustable DC power supply *V*_DC_ to achieve optimal voltage gain for maximum efficiency tracking, without parameter identification (*M*, *R*_L_), simplifies the system hardware design and control difficulties, and is suitable for the scenes of real-time changes in coil spacing and load.

### Simulation and experimental verification

#### Simulation verification

**Figure 9:**
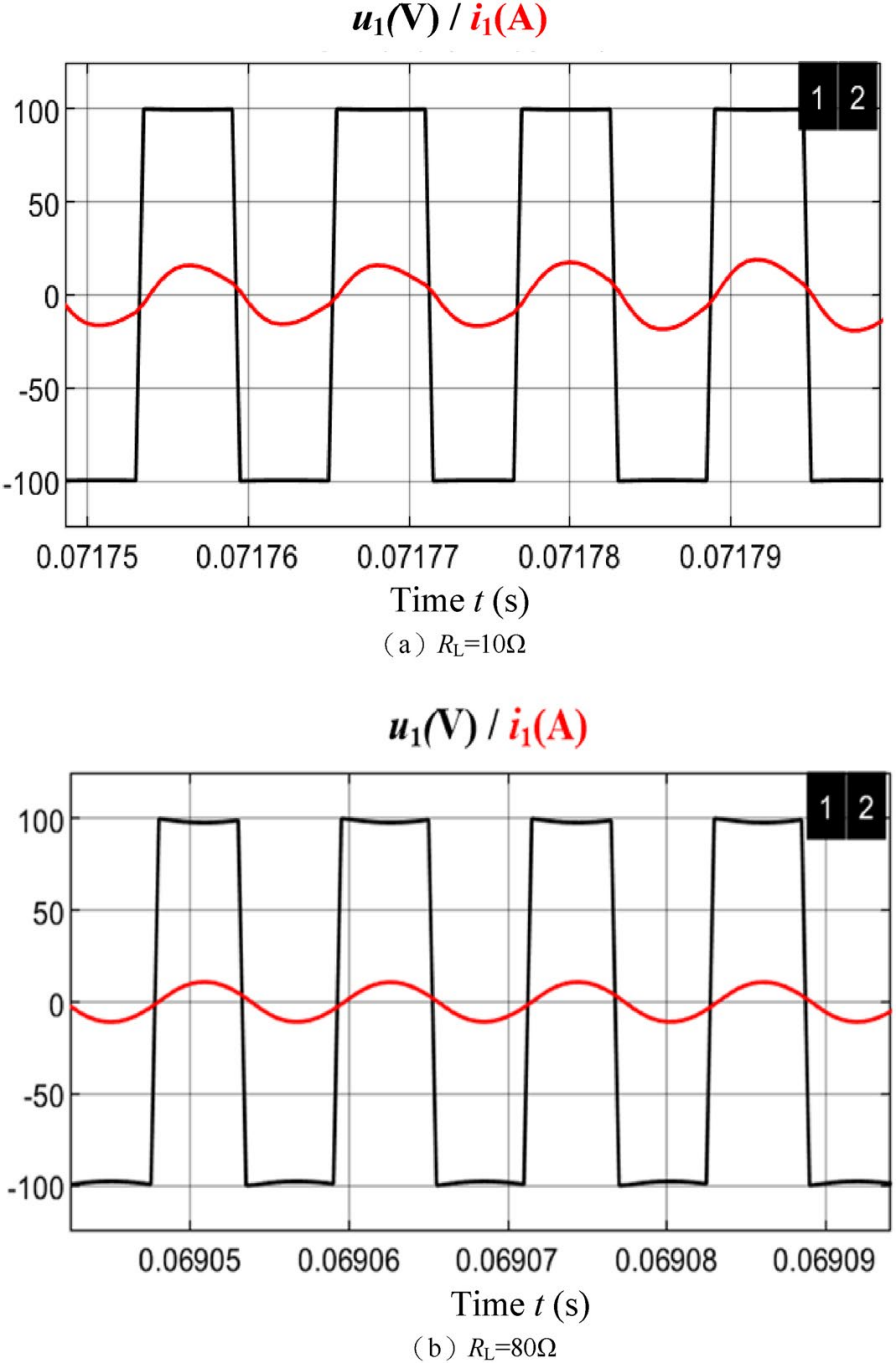
Inverter output voltage and current waveform.

In order to verify the correctness of the theoretical analysis in this paper, a SS-WPT system platform with an adjustable DC power supply and a secondary-side cascaded Boost converter is built through Simulink, and the resonator parameters are shown in Table 1. Under the premise of realizing soft switching within the range of load variation, |*μ*| should be as small as possible so that the maximum efficiency *η*_max_ of the resonator can be as large as possible. As seen in Fig. 9, when the detuning rate *μ* = − 0.06 (*C*_2_ = 36.8 nF)and the equivalent resistance *R*_e_ varies between [10,100) Ω, the system is in a weak inductance state, and the system impedance angle *φ*_1_ is between (− 15°, − 2°), which has the conditions to achieve ZVS and does not sacrifice unnecessary resonator efficiency due to excessive inductance. Figure 9 shows the waveform of inverter output voltage *u*_1_ and current *i*_1_ when the load *R*_L_ = 10 Ω and *R*_L_ = 80 Ω, the inverter output current *i*_1_ both lag slightly behind the output voltage *u*_1_, and the resonant network is weakly inductive, thus realizing soft switching.

Substituting *μ* = − 0.06 into Eq. (21), the optimal equivalent resistance ${\mathop R\limits^{*}}_{{{\text{e-opt}}}}$ can be obtained:

34 $$ {{\mathop R\limits^{ * }}}_{{{\text{e-opt}}}} = - {149}{\text{.56}} * \left( { - \;0.06} \right)\;{ + }\;{14}{\text{.15}}\;=\;{23}{\text{.124}}\;(\Omega ) $$

**Figure 10:**
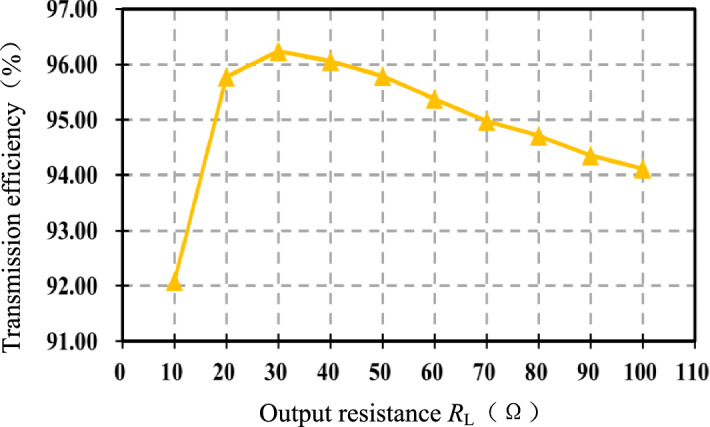
Simulation efficiency without impedance matching.

The corresponding optimal load *R*_L-opt_ = 28.5 Ω. Figure 10 shows that when the system has no impedance matching, the simulation efficiency reaches the maximum near the load of 30 Ω, which further verifies the optimal load theory.

**Figure 11:**
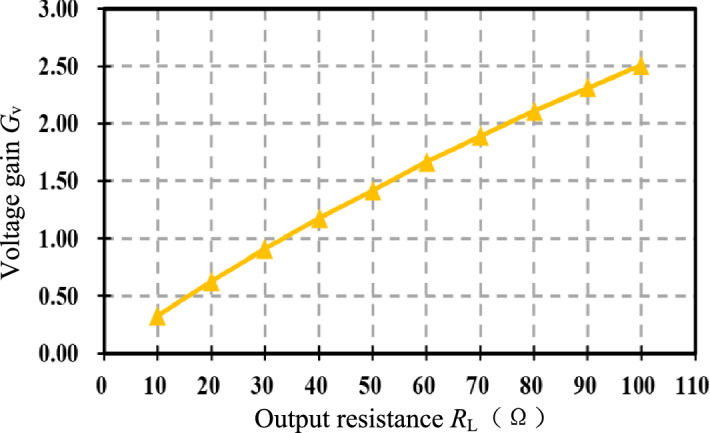
Variation of resonator voltage gain with load value.

Figure 11 shows that the voltage gain *G*_v_ of the resonator increases approximately linearly with the load *R*_L_. When the optimal load *R*_L-opt_ is 28.5 Ω, the voltage gain is 0.87.

However, substituting the detuning rate *μ* = − 0.06 into the voltage gain model Eq. (25), the optimal voltage gain is

35 $$ {\mathop G\limits^{ * }}_{{{\text{v-opt}}}} =\left[ {{1} - {10}{\text{.5695}} * \left( { - 0.06} \right)} \right] * \sqrt {\frac{0.3}{{0.78}}} \approx 1 $$

When controlling ${\mathop G\limits^{ * }}_{{{\text{v-opt}}}}$ ≈ 1, the equivalent resistance *R*_e_ ≈ 26Ω in simulation, and the matching error is about 3 Ω. The error between ${\mathop G\limits^{ * }}_{{{\text{v-opt}}}}$ ≈ 1 obtained by the voltage gain model and *G*_v-opt_ = 0.87 obtained by simulation is about 0.13, which is because there are many approximations and assumptions in the derivation of the voltage gain model. In order to make the impedance matching more accurate, the optimal voltage gain *G*_v-opt_ = 0.87 obtained by simulation is used to formulate the indirect impedance matching strategy. Consider the boundary condition (33) of the system control, where a compound control strategy is applied to the system when *R*_L_ ≥ 28.5 Ω and varies between [30, 100] Ω. Define the equivalent resistance matching deviation rate *δ*:

36 $$ \delta =\frac{{{\mathop R\limits^{ * }}_{{{\text{e-opt}}}} - R_{{\text{e}}} }}{{{\mathop R\limits^{ * }}_{{{\text{e-opt}}}} }} $$

**Figure 12:**
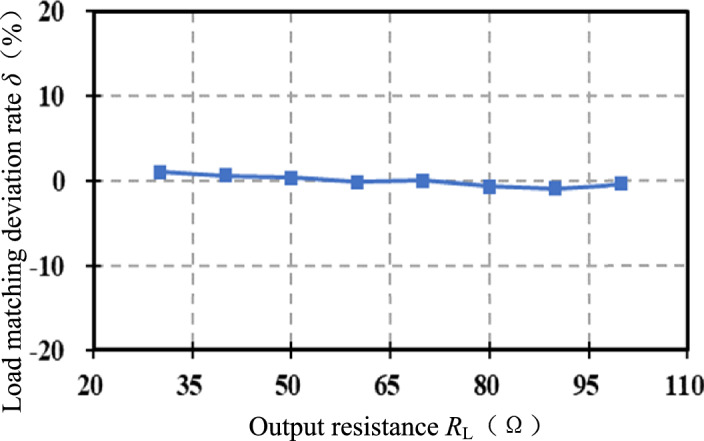
Load matching deviation rate when *R*_L_ changes.

Figure 12 shows the variation curve of the equivalent resistance deviation rate *δ* with the output resistance *R*_L_. The maximum matching error is 0.23 Ω, |*δ*| < 0.01, and the matching accuracy is high.

**Figure 13:**
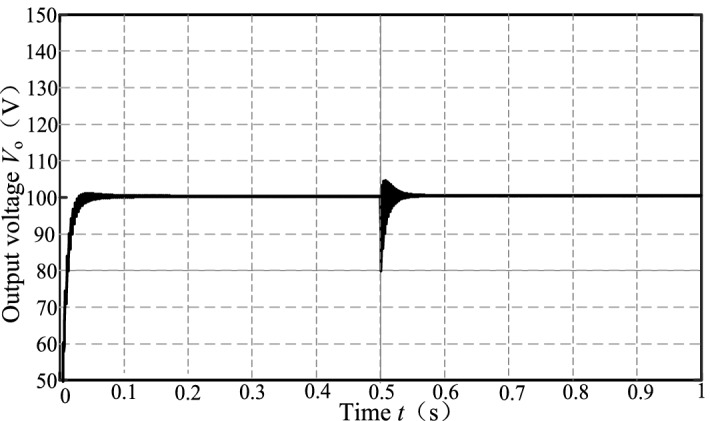
Output voltage stabilized waveform.

The output voltage waveform when the load changes suddenly is shown in Fig. 13. Under the compound control, when t = 0.5 s, the *R*_L_ suddenly drops from 80 to 40 Ω, *V*_o_ can be stabilized to 100 V again after 0.03 s with only 4% overshoot and the steady-state error is approximately 0, and the peak-to-peak output voltage ripple is 2 V.

**Figure 14:**
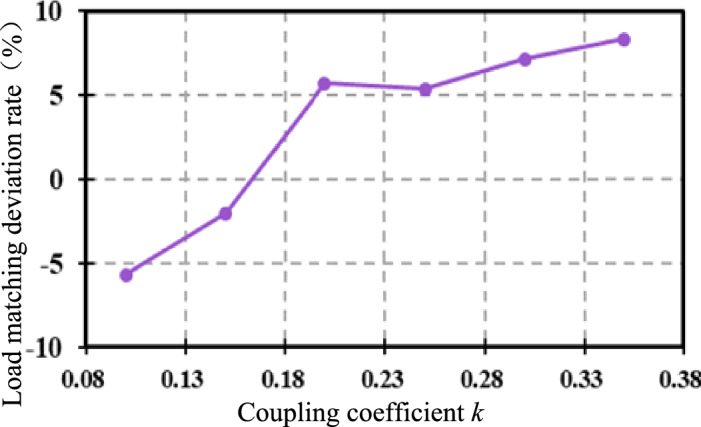
Resistance matching deviation rate when *k* changes.

The above are the simulation verifications when the coupling coefficient *k* is fixed (*k* = 0.31) and the output resistance *R*_L_ is varied. Now we verify the control effect when *R*_L_ = 80 Ω and *k* is varied between (0.1, 0.35) at 0.05 intervals (the mutual inductance *M* is varied between (16 μF, 52.5 μF). Under different *k*, the deviation rate *δ* of the equivalent resistance *R*_e_ from the optimal value ${\mathop R\limits^{*}}_{{{\text{e-opt}}}}$ is shown in Fig. 14. The average deviation rate is 0.07, which shows that the matching effect is good when *k* varies widely.

**Figure 15:**
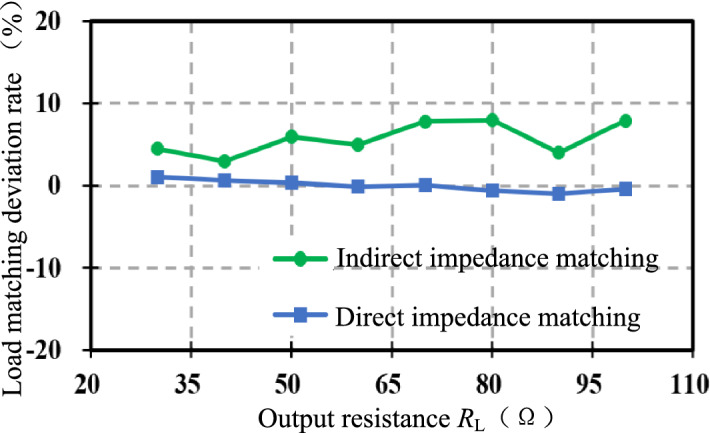
Comparison of load matching deviation rate.

Figure 15 shows the comparison curves of the equivalent load deviation rate *δ* under two control strategies. The output resistance *R*_L_ varies between [30,100] Ω. The green line is the deviation rate curve after direct impedance matching, and the blue line is the deviation rate curve after indirect impedance matching. Obviously, the accuracy of indirect impedance matching is higher, and the equivalent resistance *R*_e_ is closer to the optimal equivalent load value ${\mathop R\limits^{*}}_{{{\text{e-opt}}}}$.

#### Experimental verification

Based on the system control loop diagram given in Fig. 8 and the parameters shown in Table 1 (*C*_2_ is designed to be 36.8 nF when *μ* = − 0.06), the experimental platform shown in Fig. 15 was built to verify the effectiveness of the proposed compound control method.

**Figure 16:**
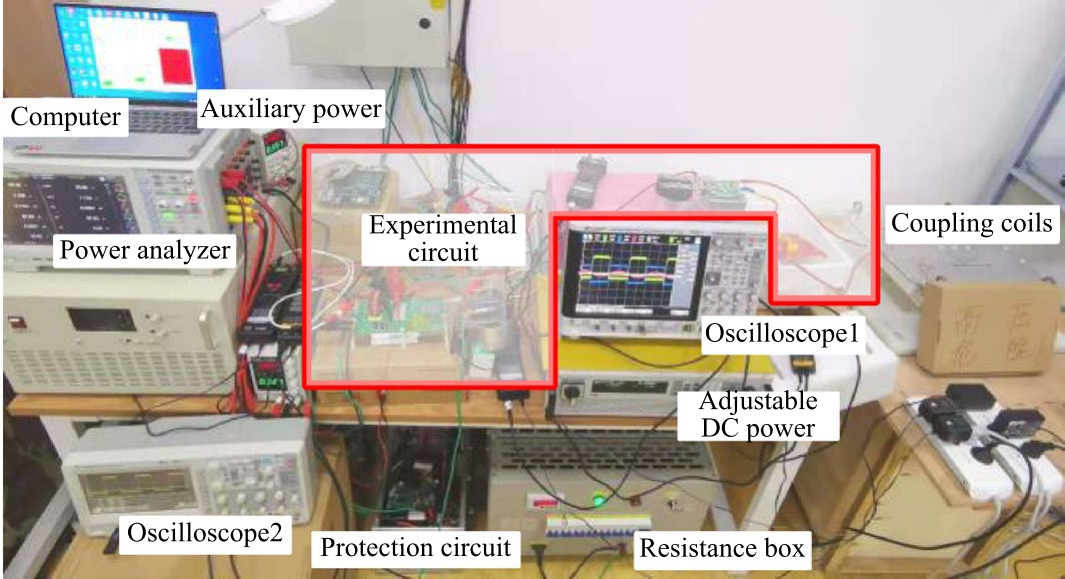
Experimental platform.

In Fig. 16, the DC power supply model IT6006C of ITECH is used as an adjustable DC source. The load adopts a power resistance box of model RXF-DC400V 10 kW. The control chip is selected as TMS320F28335 DSP. The PA5000H power analyzer is used to measure the power and efficiency of the DC–DC terminal, that is, the overall system efficiency from the input adjustable DC power supply to the output DC load. The system loss includes the loss of the inverter, resonator, rectifier, and Boost converter. Using two oscilloscopes to record the inverter output and Boost converter input waveforms, respectively. The transmission coil is wound with Litz wire and and the distance between the coils is fixed at 10 cm (*k* = 0.31).

**Figure 17:**
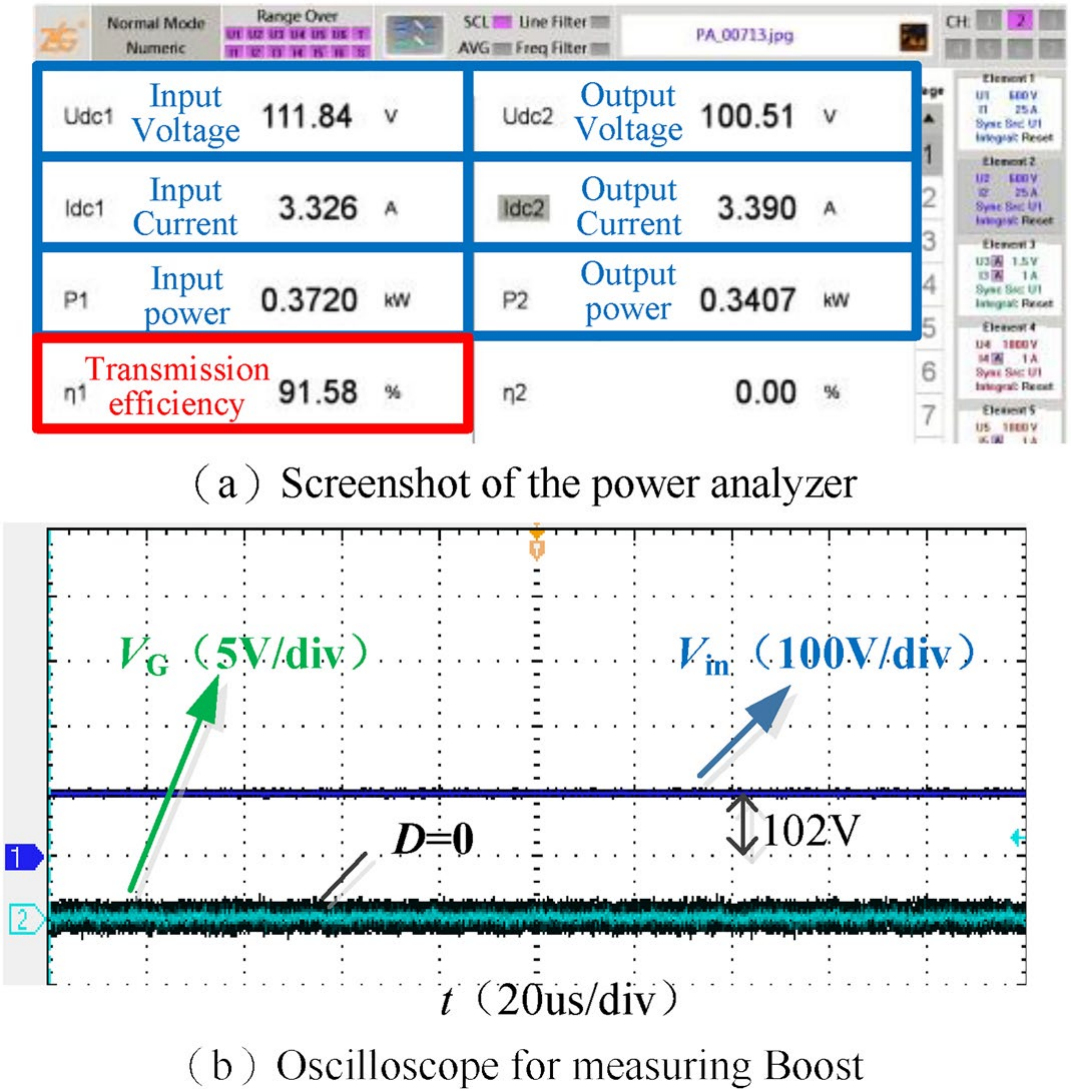
Main data and waveforms without compound control when *R*_L_ = 29.6 Ω.

According to Eq. (34), the optimal equivalent resistance ${\mathop R\limits^{*}}_{{{\text{e-opt}}}}$ of system is 23.124 Ω, and the corresponding optimal output resistance *R*_L-opt_ is 28.5 Ω. According to Eq. (35) the optimal voltage gain ${\mathop G\limits^{ * }}_{{{\text{v-opt}}}}$ obtained by modeling is 1, and the optimal voltage gain after simulation modification is 0.87. For the convenience of comparison, the Boost duty cycle *D* is set to 0 and the adjustable DC power supply *V*_DC_ is regulated to make the system output voltage 100 V in the no-compound control experiment. When the output resistance *R*_L_ is 29.6 Ω, the adjustable DC power supply *V*_DC_ is 111.84 V, the Boost input voltage *V*_in_ is 102 V, and the voltage gain is 0.91. The simulation and experiment have good consistency. Figure 17 shows the screenshot of the power analyzer when *R*_L_ = 29.6 Ω, and the waveforms of Boost input voltage *V*_in_ and duty cycle *D*.

**Figure 18:**
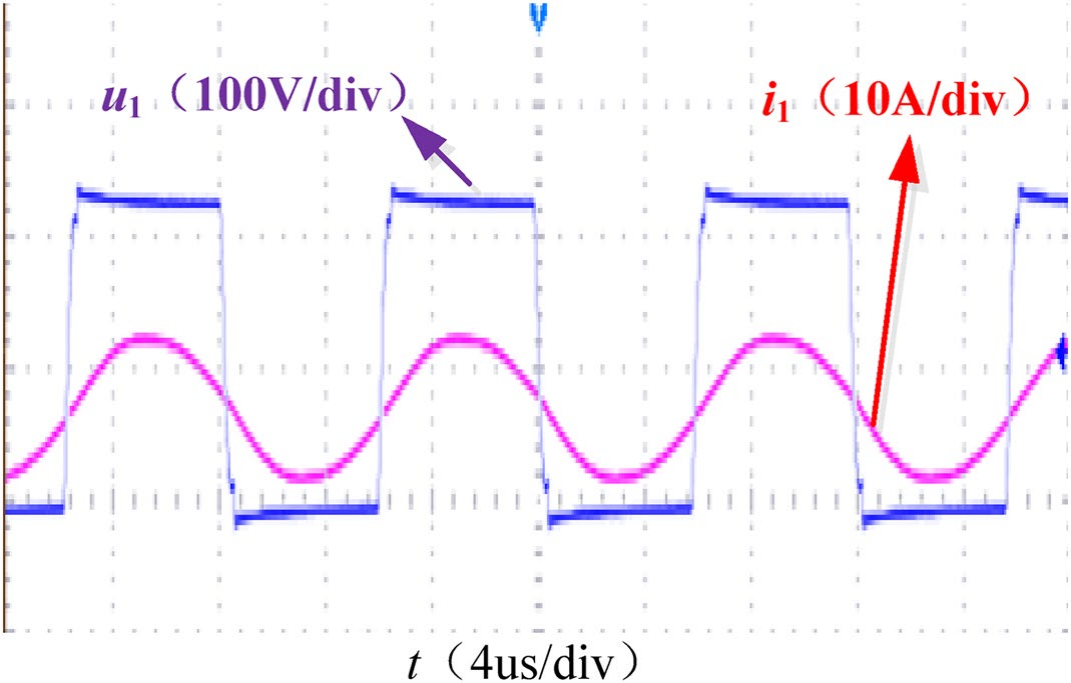
Inverter soft switching waveform.

Figure 18 shows the output voltage *u*_1_ and current *i*_1_ waveforms of the inverter. The system is weakly inductive and has the conditions to realize soft switching.

**Figure 19:**
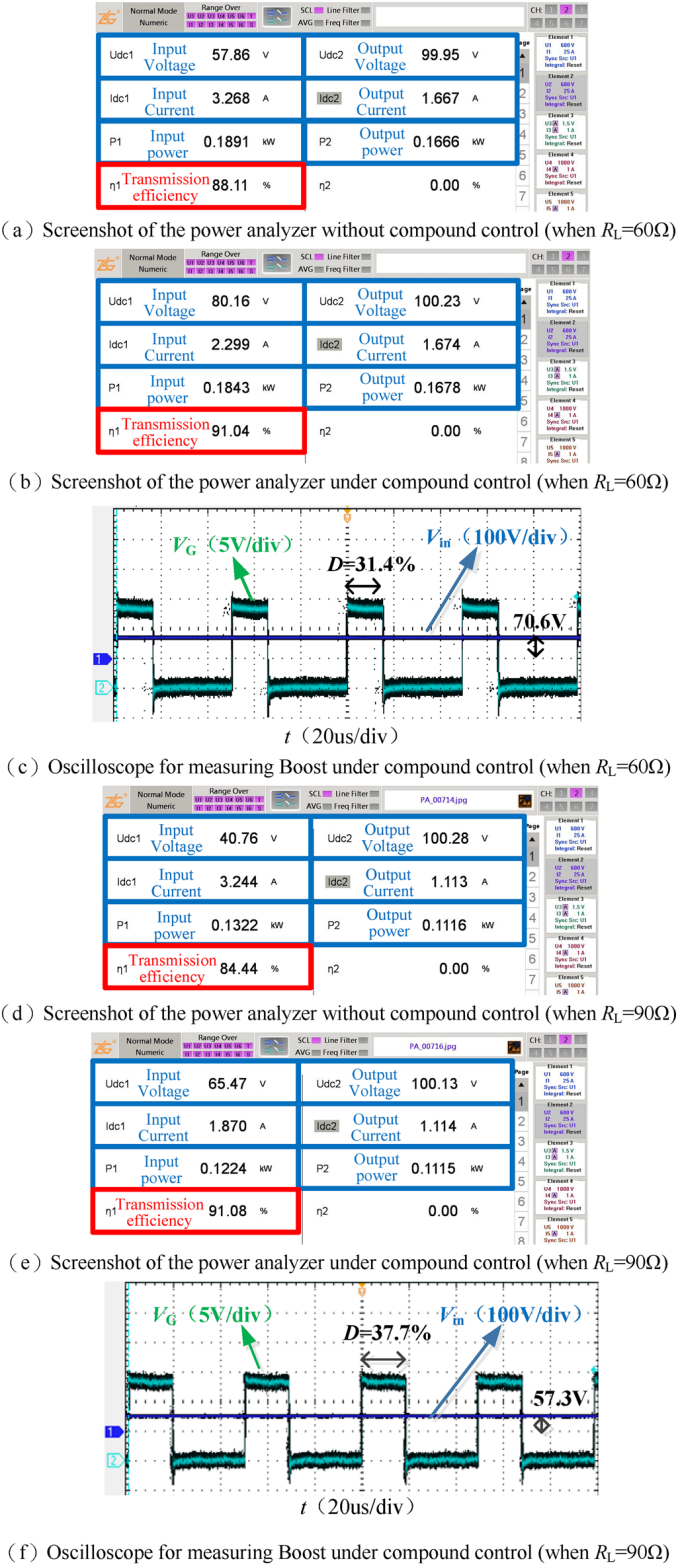
Main data and waveforms when *R*_L_ = 60 Ω, *R*_L_ = 90 Ω.

Figure 19 shows the screenshots of the power analyzer and the waveforms of Boost input voltage *V*_in_ and duty cycle *D* under compound control when the output resistance *R*_L_ = 60 Ω as well as *R*_L_ = 90 Ω. Under the system 100 V constant voltage control, the optimal voltage gain of 0.87 is used for maximum efficiency tracking.

It can be seen from Fig. 19b and c that under compound control, when *R*_L_ = 60 Ω, the output voltage *V*_o_ is stable at 100.23 V, while the adjustable DC source *V*_DC_ is 80.16 V, the Boost input voltage *V*_in_ is 70.6 V, and the voltage gain is 0.88. Comparing the screenshots Fig. 19a and b of the power analyzer, the efficiency of the DC–DC terminal has increased from 88.11 to 91.04% after the compound control.

**Figure 20:**
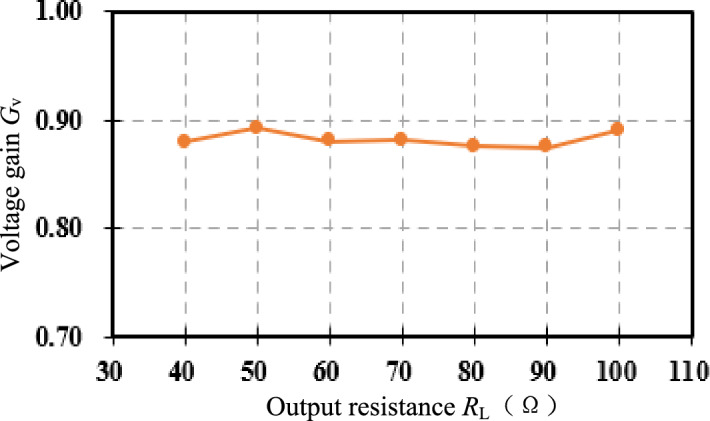
Voltage gain under compound control.

Similarly, as seen in Fig. 19e and f, under compound control, when *R*_L_ = 90 Ω, *V*_o_ = 100.13 V, *V*_DC_ = 65.47 V, *V*_in_ = 57.3 V, and the voltage gain is 0.88, Comparing the screenshots Fig. 19d and e of the power analyzer, the efficiency of the DC to DC terminal has increased from 84.44 to 91.08% after the compound control. Under compound control, when *R*_L_ changes from 60 to 90 Ω, compare Fig. 19b and e, the adjustable DC source *V*_DC_ drops from 80.16 to 65.47 V; compare Fig. 19c and f, Boost duty cycle *D* rises from 31.4 to 37.7%. Figure 20 shows the voltage gain of the resonator when the output resistance *R*_L_ changes between [40, 100] Ω under compound control, fluctuating between 0.87 and 0.9.

**Figure 21:**
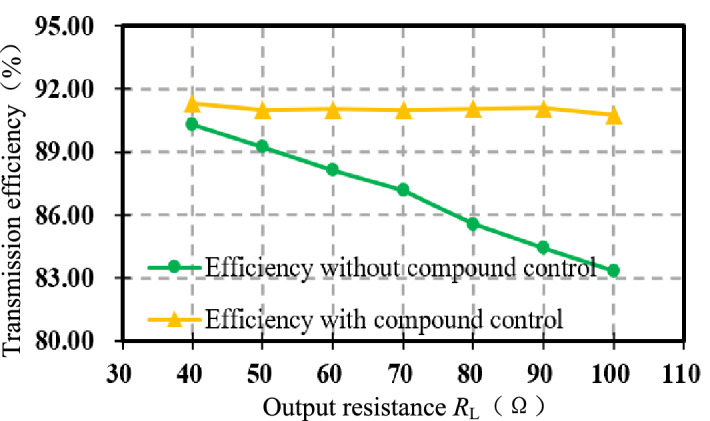
Transmission efficiency comparison chart.

Figure 21 shows that the efficiency of the DC–DC terminal before and after the compound control when the output resistance *R*_L_ is varied between [40, 100] Ω. The more the *R*_L_ deviates from the optimal value, the more significant the efficiency improvement is. When *R*_L_ = 100 Ω, the efficiency of the system is optimized from 83 to 90.73% after the compound control, an improvement of 7 points, verifying the effectiveness of indirect impedance matching using voltage gain.

**Figure 22:**
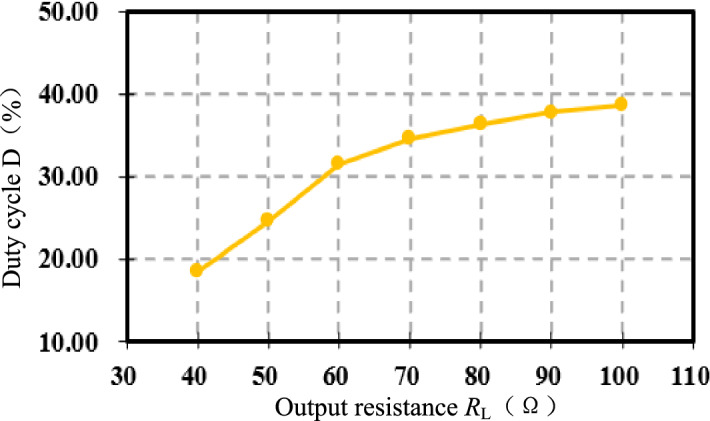
Boost duty cycle *D* under compound control.

Figure 22 shows the duty cycle *D*_∞_ of the Boost converter after compound control stabilization. The output resistance *R*_L_ increases, the corresponding *D*_∞_ increases from Eq. (31), the experimental results are consistent with the theoretical analysis.

**Figure 23:**
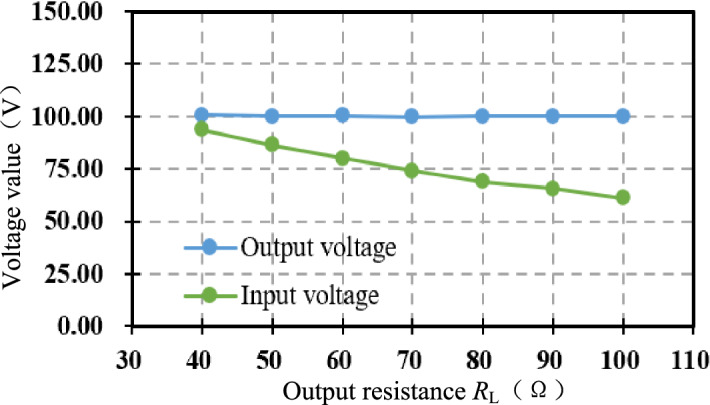
Output voltage and adjustable input voltage under compound control.

Figure 23 shows the output voltage *V*_o_ and the adjustable DC power supply *V*_DC∞_ after compound control stabilization. Under various loads, the output voltage *V*_o_ is stable at 100 V. As *R*_L_ increases, *D*_∞_ increases, the output voltage *V*_o_ is fixed when the adjustable DC source *V*_DC∞_ from the Eq. (32) becomes smaller, the experimental results are consistent with the theoretical analysis.

## Conclusion

Aiming at the problem that conventional direct impedance matching method requires mutual inductance and load identification, which makes the system complicated, this paper proposes an optimal voltage gain modulation without parameter identification to indirectly achieves impedance matching. The main contributions are as follows.

1. A single-sided detuning design method used to achieve soft switching of the inverter is analyzed. This paper adopts a secondary side detuning design with better soft switching characteristics and secondary current characteristics.
2. The principle of maximum efficiency tracking is analyzed, and the optimal equivalent resistance *R*_e-opt_ and the optimal voltage gain *G*_V-opt_ are derived when both the soft switching of the inverter and the impedance matching of the resonator are realized. The optimal voltage gain *G*_V-opt_ is only related to the fixed parameters of the resonator (*R*_1_, *R*_2_, *μ*), thus obtaining a method for maximum efficiency tracking without parameter identification.
3. Based on the secondary-side cascaded Boost converter and the adjustable DC power supply, this paper proposes a compound control strategy for a series-series-compensated topology with dual-side power control to improve efficiency and stabilize the output voltage. The dynamic coordination process of indirect impedance matching control is described in detail, and the control premise (decoupling condition of two control degrees of freedom), control result (steady state value) and control effect are analyzed.

The experimental results show that when the optimal voltage gain *G*_v-opt_ = 0.87 is utilized to achieve indirect impedance matching, the efficiency of system remains around 91% when the output resistance *R*_L_ is varied between [40, 100] Ω. The optimal output resistance *R*_L-opt_ = 29.6 Ω, when *R*_L_ = 100 Ω, the efficiency is improved by nearly 7%. Therefore, the control method proposed in this paper is feasible and effective, especially suitable for the scenarios of real-time changes in coil spacing and load.

## Data Availability

The datasets used and/or analysed during the current study available from the corresponding author on reasonable request.
